# A study protocol of a comparative mixed study of the T‐Control catheter

**DOI:** 10.1002/bco2.313

**Published:** 2024-01-02

**Authors:** José Medina‐Polo, Ana Belén Salamanca‐Castro, Yolanda Ramallo‐Fariña, Max Mòdol‐Vidal, Cristina Valcárcel‐Nazco, Clara Armas‐Moreno, Lilisbeth Perestelo‐Pérez, Lidia García‐Pérez, Miguel Ángel García‐Bello, Manuel Luque‐González, Marta Serrano‐Muñoz, Santiago Pérez‐García

**Affiliations:** ^1^ Urology Service 12 de Octubre University Hospital Madrid Spain; ^2^ Canary Islands Health Research Institute Foundation (FIISC), Evaluation Unit (SESCS) Canary Islands Health Service Tenerife Spain; ^3^ Canary Islands Research Group (Canary Islands Health Service) Network for Research on Chronicity, Primary Care, and Health Promotion (RICAPPS) Tenerife Spain; ^4^ Scientific Department Rethink Medical SL Las Palmas de Gran Canaria Spain; ^5^ Clinical Psychology, Psychobiology and Methodology Department University of La Laguna (ULL) Tenerife Spain; ^6^ InveCuid Group, Imas12 Institute 12 de Octubre University Hospital Madrid Spain

**Keywords:** CAUTI, clinical trial, Foley catheter, nursing, quality of life, urinary catheters

## Abstract

**Background:**

Foley catheters have been subject to limited development in the last few decades. They fulfil their basic function of draining urine from the bladder but cause other associated problems. T‐Control is a new silicone Foley catheter with an integrated fluid control valve whose design aims to reduce the risks associated with bladder catheterization by a multifactorial approach. The general purpose of this study is to determine the effectiveness, comfort, and experience of the patient catheterized with T‐Control® compared with patients with a conventional Foley catheter.

**Study Design:**

This trial is a mixed‐method study comprising a two‐arm, pilot comparative study with random allocation to T‐Control catheter or traditional Foley catheter in patients with long‐term catheterization and a study with qualitative methodology, through discussion groups.

**Endpoints:**

The comfort and acceptability of the T‐Control® device (qualitative) and the quality of life related to self‐perceived health (quantitative) will be analysed as primary endpoints. As secondary endpoints, the following will be analysed: magnitude and rate of infections (symptomatic and asymptomatic); days free of infection; indication of associated antibiotic treatments; determination of biofilm; number of catheter‐related adverse events; use of each type of catheterization's healthcare resources; and level of satisfaction and workload of health professionals.

**Patients and Methods:**

Eligible patients are male and female adults aged ≥18 years, who require a change of long‐term bladder catheter. The estimated sample size is 50 patients. Patient follow‐up includes both the time of catheter insertion and its removal or change 4 weeks later, plus the time until the discussion groups take place.

## 1 BACKGROUND

Urinary bladder catheterization is a common healthcare procedure for therapeutic and diagnostic purposes,^1^ usually managed by nursing staff.^2^ Overall, 15.5% and 23.6% of hospitalized patients in Europe and the United States, respectively, received an indwelling bladder catheter, with higher rates for older patients, surgery departments, and intensive care units (45–79%).^3^

Urethral catheterization is the most common bladder catheterization in clinical practice.^4^ Although the available clinical evidence promotes intermittent catheterization as the first therapeutic option to preserve quality of life^5^, ^6^ and reduce long‐term complication risks,^7^, ^8^, ^9^, ^10^ frequently indwelling catheterization is required to avoid an additional burden for patients.^11^ In addition to the negative emotions of living with an indwelling catheter,^12^ complications such as urinary tract infections (UTIs), catheter obstruction, and recurrent bladder stones^13^, ^14^ might occur. Catheter‐associated UTIs are common, causing 80% of hospital‐acquired urinary infections,^8^, ^9^ increasing morbidity, mortality,^15^ and costs.^16^, ^17^ Moreover, the little scientific literature on the quality of life of patients living with urinary catheters emphasizes findings such as lack of autonomy, fear and anxiety, and self‐image concerns.^18^

Despite their wide use, limited improvements have occurred in the design and development of indwelling urinary catheters over the last few decades. It has been suggested, however, that a patient‐managed valve connected to the catheter outlet, instead of the conventional urine drainage bag, may lead to improvements,^19^ which enable voluntary and timely opening and bladder emptying by the patient and helping maintain bladder tone and capacity.^20^ This innovation could reduce bladder irritation because periodic filling would reduce contact with the catheter tip. Periodic valve‐regulated flushing might also decrease infection rates and valve blockage.^21^, ^22^, ^23^

T‐Control is a new silicone Foley catheter with an integrated valve to voluntarily control urine flow, which enables bladder filling and its conscious regulation and emptiness, thereby reducing mucosa irritation by contact of the catheter tip. This innovative valve controls urine flow by means of three different positions, from the proximal end of the tube. The “open” and “closed” positions regulate urine flow without other additional accessories required by the conventional Foley catheter. The third or “insertion” position, only available initially for the insertion manoeuvre, prevents unwanted urine leakage thanks to a specific built‐in membrane. T‐Control has an additional safety lock to prevent accidental opening of the catheter valve.

T‐Control has undergone and passed different biocompatibility studies, including the assessment of cytotoxicity, skin sensitization, intracutaneous reactivity (irritation), and acute, subacute, and subchronic systemic toxicity (necessary results to obtain the Conformité Europeéne marking). Beyond these preclinical biocompatibility studies, usability studies have also been performed to test the ease of professional handling of T‐Control. These confirm that an easier and safer insertion technique could be provided, while also being able to be performed by one person, which reduces the staffing needs currently recommended by clinical practice guidelines.^24^ Furthermore, patients' experience in the use of bladder catheters were comparatively evaluated with T‐Control and conventional catheters and recognized the added value and the intuitive and ease of use of T‐Control.^25^ Finally, in vitro studies have observed how T‐Control significantly prevented/delayed the formation and growth of biofilm during the first 5 days, compared with conventional Foley‐type catheter.^26^ In addition, another pilot clinical study is currently being carried out with the T‐Control catheter in patients with acute urinary retention (NCT05643950), for which the first results are expected to be obtained at the end of the first quarter of 2024.

This clinical trial protocol shares the methodology to make progress in regard to the process of generating evidence on the effectiveness, safety, and cost‐effectiveness of T‐Control and to improve clinical and self‐perceived health outcomes by patients, compared with conventional catheters currently used. In addition, this trial aims to generate information on the potential contribution of T‐Control to improve the sustainability and solvency of the healthcare system.

## 2 STUDY DESIGN

### 2.1 Trial design and trial setting

This trial is a mixed‐method study comprising a two‐arm, pilot comparative study with random allocation to T‐Control catheter or traditional Foley catheter in patients with long‐term catheterization and a study with qualitative methodology, through discussion groups.

For the quantitative study, the sample of patients will be recruited in the urology service of Hospital 12 de Octubre. The identification of patients eligible to participate will be carried out by members of the urology service staff. The subjects included in the quantitative study who have completed the follow‐up will be invited by the urology clinic researcher to participate in the qualitative study.

### 2.2 Recruitment

Patients eligible to participate will be identified by the health professionals responsible for the study. All those patients who require a change of long‐term bladder catheter will be invited to participate and will be interviewed in an initial visit, where the inclusion and exclusion criteria will be checked, and the patients or their relative/caregiver (if necessary) will receive information about the study, will be invited to participate in the study, and will request their consent agreement, including the subject on the study's registration sheet.

### 2.3 Random assignment

Participants will be assigned 1:1 to one of the two trial arms of the quantitative study by a local research team member using a centralized computerized randomization system (RAND2 software, The MathWorks Inc, Natick, USA, administered by the data management team, depending on the contract research organization [CRO]).

The blinded allocation sequence is concealed by the use of a centralized computerized randomization system. The personnel responsible for catheter insertion will enrol subjects on the randomization system.

The patient's identifying number will be consecutively assigned, and inclusion in the intervention group or control group will be carried out through the randomized numerical distribution generated by a random online generator.

### 2.4 Blinding

It will not be possible to blind the study arm for the trial subjects, the health professionals, the research team involved, and the monitoring team. Data analysis will be blinded to the intervention arm as well as the laboratories that will analyse the urine and catheter samples.

In the event of adverse events that may compromise the patient's safety, data may be unmasked by means of the unique code given to each participant.

## 3 ENDPOINTS

The general purpose of this study is to determine the effectiveness, comfort, and experience of the patient catheterized with T‐Control® compared with patients with a conventional Foley catheter, by evaluating patients with long‐term urinary catheters.

Primary endpoints are as follows:

- To analyse, through qualitative techniques, the comfort and acceptability of the T‐Control® device for the patient framed in the course of the disease, identifying preferences and training and information needs for the use of the device; and
- To analyse and compare the degree of satisfaction and levels of self‐perceived health‐related quality of life of patients with both types of catheters.

Secondary endpoints are as follows:

- To assess the preliminary effectiveness of T‐Control versus a common Foley catheter, by comparing the magnitude and rate of infections (both symptomatic and asymptomatic) due to the catheter among long‐term catheterized patients. Additionally, evaluate the effectiveness of T‐Control by comparing the days free of infection on the 28th day after catheterization;
- To compare the indication of antibiotic treatments associated with catheter use between the conventional Foley‐type catheter and the T‐Control device;
- To determine the biofilm formed in the catheters and identify the microorganisms that form this to evaluate its relationship with the onset of symptomatic and asymptomatic infections as well as different adverse events;
- To compare the number and relevance of adverse events related to bladder catheterization between T‐Control and the traditional Foley catheter;
- To assess the preliminary cost‐effectiveness of T‐Control versus the traditional Foley catheter from the public healthcare services perspective; and
- To measure and compare the level of satisfaction and workload perceived of health professionals with both types of catheterization by means of a questionnaire at the end of the study.

## 4 ELIGIBILITY CRITERIA

Patient inclusion criteria are as follows: (1) men or women aged >18 years; (2) patients who require a change of bladder catheter; (3) absence of symptoms compatible with infection; (4) indication of bladder catheterization for 4 weeks; (5) maintained cognitive and physical capacity for self‐monitoring of the catheter valve; and (6) signed consent agreement.

Patient exclusion criteria are as follows: (1) symptoms of current UTI; (2) use of current antibiotic treatment or in the 2 weeks prior to inclusion in the study; (3) patients with malformations in the urinary tract; (4) immunocompromised patients; (5) patients with urological cancer; (6) patients who require continuous urine drainage (in the case of patients in the study arm) or hourly urine output measurement; (7) catheter insertion requiring more than one attempt; and (8) inability to read and understand Spanish.

## 5 METHODS

### 5.1 Interventions

#### 5.1.1 Intervention arm: T‐Control catheter

The T‐control catheter is a flexible, silicone tube with an inflatable balloon at the distal tip, a polytetrafluoroethylene (PTFE) membrane integrated into its body, and a sliding fluid control valve. The valve, built into the catheter, provides additional functions to the catheter, such as turning urine flow on and off after insertion (functions currently provided by accessories such as caps or valves) and controlling urination during the insertion process (function not provided by any other device). Accidental loss of urine can thus be avoided from the first moment of use until its withdrawal. In addition, it has a safety cap that reduces the possibility of accidental valve movements before use or during transport and fixing. The design has been developed in such a way that, once inserted, it facilitates autonomous use even for the elderly or patients with limited manual dexterity. The device is sterile and single‐use, like any conventional Foley catheter.

The continuous use of a single device of T‐Control cannot exceed 30 days. The cumulative use for each type of T‐Control device can exceed 30 days.

T‐Control is manufactured, sterilized, and packaged by the subcontracted company Conod Medical Co., Ltd under the specifications of Rethink Medical and under its quality standards, as well as its accessory holder, also manufactured and packaged by the same subcontracted company.

#### 5.1.2 Control arm: Foley catheter

Silicone Foley catheters are transurethral balloon catheters used to treat bladder emptying disorders, as well as drain urine from the urinary tract, continuous fluid irrigation, and/or medication administration. It is suitable to be used for a prolonged period of no more than 29 days and in Urology, Internal Medicine, Surgery, Obstetrics, and Gynaecology Services.

For this arm, conventional two‐way silicone Foley‐type catheters will be used. They consist of a body, drainage funnel, inflation funnel, and balloon valve. The product is sterile and single use.

### 5.2 Study procedures

**FIGURE 1:**
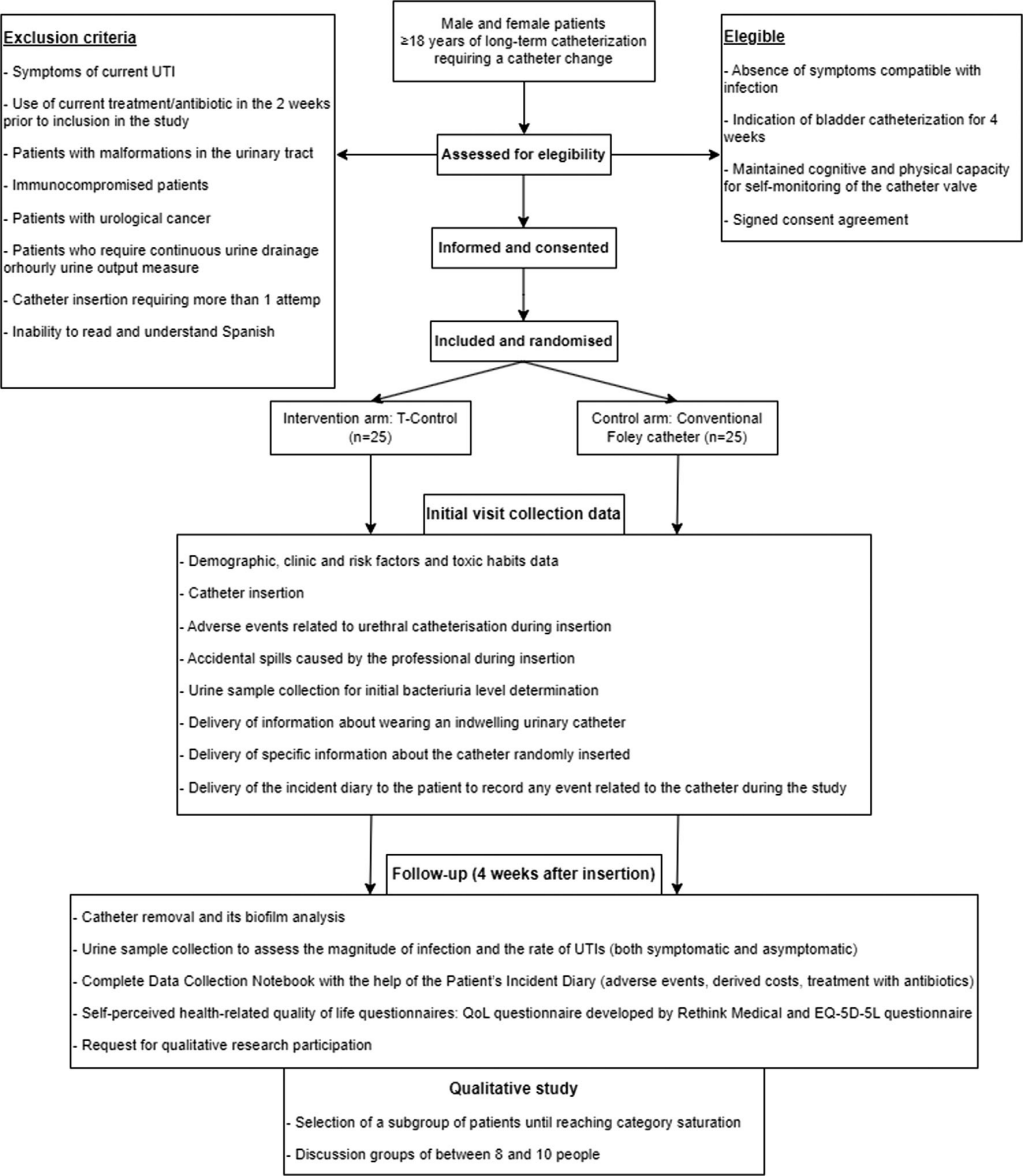
Flow diagram describing the participant timeline through the clinical trial. EQ‐5D‐5L, EuroQol questionnaire—five dimensions—five levels; QoL, quality of life; UTI, urinary tract infections.

The flowchart in Figure 1 describes the participant timeline throughout the study. The catheter will be inserted by the research staff after obtaining informed consent (Data S8), and the inclusion and randomization of the participants. The healthcare professional who inserts the catheter must have experience in bladder catheterization. Professionals without sufficient experience in catheterization involved in the study will receive specific training prior to the start of the study. In addition, the research staff will receive specific training on the device prior to the start of the study (Data S1). All the staff involved in the study will have access to the user instructions at any time.

After catheter insertion, participants and their family, friends, or other informal caregivers will receive information about wearing an indwelling urinary catheter and specific information about the device randomly inserted, the standard Foley or the T‐Control catheter (Data S2). Standard catheter care is permitted during the trial both managed by the participants or participants' caregivers. In addition, patients will receive an incident diary (Data S4) in which they can record any incident during the bladder catheterization period. Four weeks after indwelling bladder catheter insertion, patients will be contacted by the urology outpatients department for a follow‐up visit, during which the study catheter will be removed and Clinical Data Notebook completed (Data S3).

Finally, after the follow‐up visit, a patient subgroup will take part in the discussion groups to evaluate their acceptability of the device used as well as their experiences, preferences, and needs (at which point their contribution to the research will finish).

Health professionals who have participated in the study will complete at closure, and after signing the informed consent form, a survey where they will evaluate their level of satisfaction and workload perceived for both types of catheters included in the study (Data S7).

#### 5.2.1 Criteria for discontinuing or modifying allocated interventions

Reasons for going off or discontinuing the trial are as follows:

- Adverse events that may compromise the patient's safety;
- Need for antibiotic treatment for a reason unrelated to the use of a bladder catheter;
- The patient's refusal to continue (in the event that, according to clinical practice, they still needed to carry an indwelling bladder catheter, the T‐Control catheter would be exchanged for the conventional Foley‐type catheter); and
- Death.

### 5.3 Sample size determination

The sample size for the quantitative primary endpoint “degree of satisfaction and levels of self‐perceived health‐related quality of life of patients” has been calculated in terms of standardized differences through Cohen's delta. Assuming a significance level of 2.5%, given that the endpoint is composed of two different questionnaires (EuroQol‐5D‐5L [EQ‐5D‐5L] and Catheter‐related quality of life questionnaires), and expecting large differences (0.95) between groups, a sample size of 25 per group would be enough, taking into account a dropout rate of 5%.

Regarding the qualitative primary endpoint, it is expected to reach information saturation with a maximum of four discussion groups composed of between 8 and 10 participants (a total of a maximum of 40 subjects), so the calculated sample size is considered sufficient.

### 5.4 Methods of data collection

The source and timing of measures are summarized in Table 1. In accordance with the proposed research objectives, the outcome measures that will be performed are the following.

**TABLE 1:** Source and timing of variables obtained for the study.

Variable	Method of obtention	Data collection format	Source	Timing			
				Baseline	4 weeks	Up 6 months AFUV	Close‐out
Baseline data (sociodemographic, clinical, and life habits)	Personal interview/PCDN	Paper/electronic	P and HP	X			
Magnitude of infection	Urine culture/PCDN	Paper/electronic	Microbiology laboratory	X	X		
Rate of infection (asymptomatic and symptomatic UTI)	Urine culture/PCDN	Paper/electronic	Microbiology laboratory	X	X		
Adverse events during catheter insertion	Observation/PCDN	Paper/electronic	HP	X			
Adverse events during catheterization time	ID/PCDN	Paper/electronic	P		X		
Catheter biofilm	Viable cells counting/PCDN	Paper/electronic	Microbiology laboratory		X		
Antibiotic treatments related to catheterization	ID/PCDN	Paper/electronic	P and HP		X		
Healthcare resources utilization	ID/PCDN	Paper/electronic	P and HP		X		
Quantitative data for QoL and catheter satisfaction	Patients' questionnaire and EuroQoL 5D questionnaire	Paper/electronic	P	X	X		
Qualitative findings for acceptability and experience	Discussion groups	Audio recording	P			X	
Catheter opinion (professionals)	Professional questionnaire	Paper/electronic	HP				X
Workload perceived	NASA‐TLX questionnaire	Paper/electronic	HP				X

Abbreviations: AFUV, after follow‐up visit; EuroQoL 5D, EuroQol questionnaire—five dimensions—five levels; HP, health professional; ID, incident diary; NASA‐TLX, NASA Task Load Index questionnaire; P, participant/relative, friend, or informal carer‐completed questionnaire; PCDN, Patient Clinical Data Notebook; QoL, quality of life perceived; UTI, urinary tract infections.

#### 5.4.1 Primary outcomes

##### Acceptability of the T‐Control device as well as the patient experience framed within the course of the disease; identification of the preferences and needs for training and information for use of the device and possible future improvements for the T‐Control device

The discussion group technique will be used in order to identify the discrepancies or affinities that exist in the participants' discourses, and therefore, it will be very useful to identify the aspects that the participants consider most and least beneficial based on their experience. The discussion groups will be moderated by a researcher with experience in this field who does not have a therapeutic relationship with the participants, thus allowing the free expression of their opinions. The discussion groups will be recorded with a voice recorder (and if the participants approve it, also in visual support to be able to analyse the non‐verbal language in more detail) and will be transcribed in its entirety verbatim for later analysis. For the analysis of the information, not only what is said but also the sounds and paralinguistic elements of the language will be considered, in order to collect the contextual information in which the opinions are expressed. The analysis of the information will be carried out concomitantly with its collection (from the narratives collected in the recordings) through axial coding because the categories, subcategories, and families of codes will be related to each other in order to find the relationships between the categories, their properties, and their dimensions. A software will be used for the qualitative analysis of narrative data to help in coding the data and relating codes with other properties, both numerical and categorical. The criteria of investigator triangulation, credibility, and transferability will be applied in order to ensure the validity of the data obtained.

##### Self‐perceived health‐related quality of life

The following instruments are administered to patients:

- EQ‐5D‐5L^27^: This is a generic health‐related quality of life questionnaire that evaluates five domains: mobility, self‐care, usual activity, pain/discomfort, and anxiety/depression. Each domain is scored on a 5‐point scale, which yields a descriptive system that can be combined into a five‐digit number reporting the patient's state of health. Each EQ‐5D‐5L health state can be converted to a single summary index by applying a formula that attaches weights to each level in each dimension. A number of formulae or value sets are available for different countries, based on the valuation of EQ‐5D health states from general population samples. In this study, the value set estimated for Spain by Ramos‐Goñi et al.^28^ will be used. After applying these weights, the range of the summary index is 1 (perfect health) to 0 (health state equivalent to death). Negative values represent health states considered to be worse than death. The questionnaire also includes a visual analogue scale where responders are asked to indicate their health status on the day of the interview, ranging from 0 (worst possible health) to 100 (best possible health).
- Catheter‐related quality of life questionnaire: To evaluate the specific health‐related quality of life for long‐term catheterized patients, Rethink Medical has developed two specific instruments created in the context of this project based on the experience of our previous studies (Patient's workshop).^25^ The first one (Data S5), administered during the initial visit, consists of 47 questions, related to the patient's experience and journey with catheterization before the study, as baseline data. The questions are related to the type of catheter and accessories used, the experience of catheterization, emotions felt at the beginning (right from the moment of the prescription), satisfaction and comfort with the catheter, complications associated with catheter use, usability and complications associated with accessories and changes in the personal habits caused by the catheter's use, and how these issues have affected their lives. The second one (Data S6), administered during the follow‐up visit, consists of the same questions as the first one (but referred to the catheter and accessories used during the study) plus four questions related to information received when catheterized for the first time (total 51 questions). In order to quantitatively evaluate the answers, statements will include single answers with scores on a scale from 0 to 10, 0 to 5, and 0 to 4 for both questionnaires. The questionnaire devised by Rethink Medical is expected to be validated during the clinical study in conjunction with another study specially designed for its validation.

#### 5.4.2 Secondary outcomes

##### Magnitude of infections

The magnitude of infection will be obtained from the analysis of urine culture samples taken from patients twice, during inclusion (time 0) and during withdrawal of the catheter (time 4 weeks; Table 1). In the event that the patient is prescribed an antibiotic for a UTI before the 28th day, the magnitude of infection will be evaluated at the time of prescription, through the analysis of a urine culture sample, according to usual clinical practice. The research staff will take between 5 and 10 mL of urine sample in a sterile container which will be transported to the laboratory in the shortest time possible (<2 h). If the transport or processing cannot be performed immediately, the sample will be refrigerated between 2°C and 8°C without exceeding 24 h until processing. The second urine sample will be collected from the new catheter inserted at the follow‐up visit. After seeding the urine in specific culture media, the microorganisms that have grown in the culture will be analysed to determine the number and type of microorganisms present using techniques based on biochemical tests and Matrix‐Assisted Laser Desorption/Ionization Time‐Of‐Flight (MALDI‐TOF) type mass spectrometry.

##### Rate of symptomatic and asymptomatic infections

The magnitude of infection obtained from the urine samples will be classified according to the following criteria based on the European Association of Urology (EAU) clinical guidelines^29^:

- Symptomatic infection: The presence of pathogenic microorganisms in amounts greater or equal to 1000 Colony Forming Units/mL (CFU/mL) accompanied with symptoms will determine the existence of the infection.
- Asymptomatic infection: In the absence of infection symptoms, a quantity of microorganisms greater than or equal to 100 000 CFU/mL will indicate asymptomatic infection.

In addition to the presence or absence of infections to assess their rate, this variable will also be collected using the outcome measure “infection‐free days” (with a maximum possible value of 28 days) to be compared between Foley‐type catheters and T‐Control. Suppose the patient at some point prior to the follow‐up visit presents symptoms compatible with UTI. In that case, a urine culture will be performed to confirm the presence or absence of urinary infection, according to usual clinical practice. In the event that the patient receives an antibiotic for a UTI while he/she takes part in the study prior to the follow‐up visit, this will be recorded in the medical record. However, the patient will also be asked about it at the follow‐up visit when he hands in his/her incident diary, and this will be counted as an infection.

##### Indication of antibiotic treatments associated with catheter use

At the end of the follow‐up visit, antibiotic treatments will be recorded along with the dose and treatment time.

##### Determination of the biofilm formed in the catheters and identification of the microorganisms present

The catheter removed will be sent to the laboratory, and with the help of sterile gloves and scissors, a 1 cm‐sized fragment corresponding to the part below the balloon will be cultured for each catheter to assess whether biofilm is present or absent. The microorganisms forming the biofilm will be quantified as CFU per catheter piece. These microorganisms will also be identified by means of MALDI‐TOF‐type mass spectrometry to establish statistical relationships with those identified in the urine cultures. In the event that during the course of the 14 days in which the patient will be catheterized, he receives antibiotic treatment due to a UTI, this will be taken into account when analysing the results obtained in the catheter's biofilm determination.

##### Number of adverse events related to catheterization

The type and number of adverse events will be registered in the Patient's Clinical Data Notebook: accidental disconnection of the catheter, obstruction, pain, loss of urine per catheter, haematuria, and accidental spills caused by the professional during insertion. The patient will receive a Patient Incident Diary to write down the incidents, along with other information that may be of interest during catheterization. Timing and source of adverse events data are summarized in Table 1.

##### Healthcare resource use

The costs arising from catheterization, such as the consumption of consumable materials and resources and diagnostic tests (urine cultures and catheter cultures and biofilm analysis), will be collected in the Patient's Clinical Data Notebook and will be evaluated from the public healthcare services perspective. The analysis will also include costs because of patient contacts with primary care services, hospital admissions and length of stay, outpatient visits, emergency attendance, and medications prescribed during the study period. The information related to healthcare resource use will be drawn from each patient's electronic clinical record.

##### Level of satisfaction and workload of health professionals

The following instruments are administered to health professionals:

- NASA Task Load Index^30^: This is a subjective, multidimensional, and widely used evaluation tool that qualifies the perceived workload to evaluate the effectiveness of a task, system, equipment, or other performance aspects. The questionnaire evaluates six dimensions (mental, physical and temporal demand, performance, effort, and frustration) which enables rating them on a 1 to 10 scale, 1 being the lowest score and 10 the highest score.
- Health professional satisfaction questionnaire: The questionnaire is specifically devised and based on the experience of previous LivingLab studies^24^ to quantitatively measure satisfaction with the devices used by health professionals (conventional Foley and T‐Control catheters). This questionnaire includes an initial section with 12 statements regarding the catheter insertion process. Health professionals will rate these statements for both devices used during the clinical trial on a 1 to 5 scale according to whether or not they agree with the statements. The second section is intended for health professionals to make a comparison between both devices by means of 11 statements for which they will have to indicate which device best fits these statements according to their opinion. They can only choose one device for each statement. Finally, the questionnaire consists of a free section in which health professionals can write any comments or suggestions. The usability of the device by professionals will also be also tested, verifying that the packaging is adequate and the instructions are understandable and contain all the information necessary to use the device safely.

Baseline data for subjects such as sociodemographic (age, sex, education level, marital status, coexistence, and type of health system user) as well as clinical (diagnostic, symptoms, and previous use of bladder catheter and its accessories) and risk factors and toxic habits data (smoking habits, alcohol consumption, and sedentary lifestyle) were collected.

### 5.5 Data management

The data will be stored in a secure computer database and kept confidential in accordance with Europe Union and Spanish Data Protection Legislation (General Data Protection Regulation and the Data Protection Act 2018). Personal data are not kept longer than necessary for the purpose for which it is processed. Access rights to the data set are managed. The principal investigator of the centre and the research coordinator will have access to the full data set to enable analysis at the trial end, while the trial statistician, who is part of the CRO, will only have access to the participants' code. Authorized representatives or competent authorities will gain access to those portions of the medical records relevant to the clinical research by means of cross‐reference with clinical research personnel to verify the data if required. All computerized data will be identified solely by a code. All essential data and documents will be kept for a period of at least 10 years after the trial ends.

The personnel in charge of monitoring will enter the data collected by the research team at the centres into the study database. Safety data, case report forms, and subject questionnaires, as well as biological sample results, will be entered into the database. Monitoring staff will work closely with the research teams at the centres to ensure that the data are as complete and accurate as possible. Data quality is improved by means of a wide range and consistency checks included in the monitoring activities.

The discussion groups will be performed by researchers with experience in qualitative research. The data will be added to the database by the staff in charge of monitoring.

### 5.6 Oversight and monitoring

An independent CRO will be in charge of monitoring the clinical trial. This CRO includes staff with clinical, statistical, and methodological expertise. The Sponsor will meet with the CRO after every monitoring visit at the centre to ensure the trial is executed and carried out properly, make recommendations, and report any event that has to be passed on to the Ethics Committee or the competent regulatory health authorities.

There are no scheduled interim analyses for efficacy or futility. However, during the clinical study, the external monitor will have regular contact with the centres where the study is performed. These contacts will include visits to confirm that the facility is in accordance with specified standards and that the clinical research teams are performing the procedure as directed, as well as trial progress and any safety issues.

Retention of subjects is promoted by regular contact with the staff responsible for the study and ensuring adequate outcome measures collection. All data collected are retained and used in the analysis. Data from subjects who terminate the study earlier will also be included in a substudy analysis.

Deviations from the allocated study will be recorded in the case report forms and evaluated as a secondary endpoint.

### 5.7 Analysis plan

A statistical analysis plan will document the scheduled analysis, to be finalized before the data lock. The final analysis will take place after full recruitment and follow‐up, at the end of the study.

The analysis population will consist of all patients who meet the selection criteria and are randomized. All patients included in the study and excluded from the analysis will be listed along with their reason for exclusion. Additionally, additional analysis will be performed on patients who complete 4 weeks with the catheter. Subjects who withdraw from the study for any reason after signing the informed consent will not be replaced. No imputation will be made for missing data.

Descriptive statistics will be carried out on the variables under study, using frequencies and percentages for qualitative variables and mean with standard deviation for quantitative variables that follow a normal distribution; otherwise, the median and interquartile ranges will be calculated.

A bivariate analysis will be carried out between the control–study groups with respect to the independent variables to know the comparability of the groups using the Chi square tests for qualitative variables and for the quantitative variables *t*‐Student or Mann–Whitney U, depending on the normality of the sample. All are under a 95% confidence interval and considering statistically significant results with a *p*‐value < 0.005.

In the event that the results are discordant, deviations and/or violations of the protocol will be analysed. For all analyses, the SPSS 24.0 statistical package^31^ will be used and will be carried out by a person who will not know if the patients have been in the intervention or control group.

#### 5.7.1 Cost‐effectiveness analysis

An economic evaluation of T‐Control versus the conventional Foley catheter from the public healthcare services perspective will be performed according to the analytical methods accepted by the scientific community.^32^ The cost‐effectiveness measure will be the incremental cost per quality‐adjusted life year (QALY) gained. QALYs are a generic health measure that combines information on life expectancy with the patient's quality of life. QALYs will be calculated based on the health‐related quality of life data collected according to the EQ‐5D‐5L instrument which will be collected for each patient. The time horizon will be the study duration. Costs included in the analysis will be those incurred by the public healthcare service. Unit costs will be obtained from the hospital centres accounting records whenever possible, from the eHealth cost database (Oblikue Consulting), and from national public sources. The mean total cost of each intervention evaluated will be presented using basic descriptive statistics (means, medians, and measures of variability such as variance).

Cost‐effectiveness will be calculated as the incremental cost‐effectiveness ratio which results from dividing the difference in costs between interventions by the difference in effects observed (QALYs). Nonparametric methods based on bootstrapped simulations will be used to calculate the confidence intervals around the incremental cost‐effectiveness ratio. In addition, the same nonparametric methods will be used to construct a cost‐effectiveness acceptability curve that will reveal the probability that each alternative is cost‐effective for different cost‐effectiveness thresholds (willingness to pay for an additional unit of effectiveness). Finally, deterministic sensitivity analysis (one‐way, two‐way, and multiway) will be performed with the aim of analysing the impact of the parameters on the cost‐effectiveness results. The analysis will be performed using the software R and the statistical package STATA (StataCorp LLC, College Station, USA).

## 6 DISCUSSION

This is the second clinical trial to investigate the new T‐Control catheter in order to provide robustness to the clinical stage of the device and obtain more data regarding it in real clinical practice. As in the first clinical study, one of the most important data to collect is the magnitude and ratio of UTIs developed in comparison with the conventional Foley‐type catheter. However, in this study, the focus is especially on the quality of life of the patients, because eligible participants (men and women) are long‐term catheterized, so this aspect of bladder catheterization has special importance for them. In this regard, the greatest limitation of the study is due to the fact that the instrument developed specifically for the study “Catheter‐related QoL questionnaire” has not yet been validated, and in fact, it is expected to be able to validate it during the course of this study in conjunction with another study specially designed for its validation.

The study could contribute to improving health (infection prevention) and social well‐being (greater quality of life, autonomy, and ability to live with the disease) among patients who use permanent bladder catheters, although it is true that the patients taking part in the study will be men and women with different clinical reasons for being catheterized, which implies a certain degree of heterogeneity in the sample.

It is important to note another of the limitations of the study is although the results analysis team will be blinded by a unique code given to each patient, neither the participants nor the health professionals, the research team involved, and the monitoring team the research team involved and the monitoring team will be blinded. However, the use in the study of both objective and subjective tools to evaluate the different aspects of bladder catheterization related to patients (infections, adverse events, biofilm formation, quality of life, and acceptance of the device), health professionals (burden work and device satisfaction), and impact on the health system (antibiotic treatment prescription and associated costs) is a strength that also should be remarked.

The study aims to promote an active and healthy lifestyle, preventing negative consequences for users. From the point of view of the work environment, the project tries to improve the working conditions of health personnel, reducing occupational risks such as spillage and contamination by contact with urine and offering an easy‐to‐use product that could make it easier to comply with bladder catheter insertion protocols without difficulty or need for additional help.

The development of this clinical study could help the sustainability of the Health System, because it offers a profitable product, with the potential to reduce infections, emergency visits (including the possible need for hospitalization), personnel costs, and so forth. Reducing the need for emergency healthcare visits, with or without admission to hospital care, is of particular interest as the Spanish healthcare system is under severe pressure, partly due to the existing shortage of registered nurses. It also aims to contribute to improving public health conditions, reducing the use of antibiotics, and mitigating the risks of transmission during pandemics such as COVID‐19.

## 7 ETHICS AND DISSEMINATION

### 7.1 Ethics

This study will be carried out in accordance with the recommendations established in the Declaration of Helsinki and the Standards of Good Clinical Practice. The Hospital Universitario 12 de Octubre Research Ethics Committee approved this study. The taking part of subjects is only contemplated for the current study, so they will not be contacted to offer them the possibility of taking part in subsequent studies. The Ethics Committee, composed of independent members, oversees the trial's conduct and progress. The SPIRIT Statement's recommendations have been followed for the drafting of the protocol and improve their completeness (Data S9).

### 7.2 Dissemination plans

A description of this clinical trial is available online (http://reec.aemps.es and https://clinicaltrials.gov/). The full protocol is available at https://clinicaltrials.gov/, and requests for participant‐level data and/or statistical code can be made in writing to info@rethinkmedical.es. The final trial data set generated and/or analysed during the current study may be available to the research coordinator and sponsor upon reasonable request.

The findings will be disseminated by means of scientific articles, national and international congresses or meetings, University Associations, both national and international, related to nursing and urology, and internal presentation or promotion for the wider community. The Sponsor adheres to the definition of authorship established by the International Committee of Medical Journal Editors.

## AUTHOR CONTRIBUTIONS

All the authors have contributed to the study design process and manuscript writing. A. B. S. C. is responsible for the design of the discussion groups qualitative analysis of the acceptability of the new T‐Control device and patient experience. C. V. N. and L. G. P. are responsible for the design of cost‐effectiveness analysis and economic evaluation. S. P. G. and M. L. G. are responsible for the conception of the study and its coordination. M. L. G., M. S. M., C. A. M., and M. M. V. are responsible for the design of the catheter's biofilm analysis protocol and the design of the self‐perceived catheter‐related quality of life questionnaire. S. P. G. is the principal clinical investigator and is responsible for the clinical coordination between centres, and Y. R. F. and M. A. G. B. are responsible for the methodological design of the clinical trial and statistical analysis plan. Y. R. F. and M. M. V. drew up the initial manuscript draft. All authors made substantial contributions to the revising of the manuscript and approved the final version.

## CONFLICT OF INTEREST STATEMENT

This group of authors J. M. P., A. B. S. C., and S. P. G. declare that they have no competing interests. This group of authors Y. R. F., C. V. N., L. P. P., L. G. P., and M. A. G. B. belongs to the Evaluation Unit (SESCS), Canary Island Health Service (SCS) (www.sescs.es), an institution of the Canary Islands Government that is also a member of the Spanish Network for Research on Chronicity, Primary Care, and Health Promotion (RICAPPS, www.ricapps.es). This article is part of the early dialogues activity carried out by the SESCS as an Evaluation agency and declares that they have no competing interests. M. L. G., M. S. M., C. A. M., and M. M. V. are part of Rethink Medical S.L., which is the Sponsor of this study, owning the rights of T‐Control.

## Supporting information


**Data S1.** Supporting Information.

**Data S2.** Supporting Information.

**Data S3.** Supporting Information.

**Data S4.** Supporting Information.

**Data S5.** Supporting Information.

**Data S6.** Supporting Information.

**Data S7.** Supporting Information.

**Data S8.** Supporting Information.

**Data S9.** Supporting Information.
