# Supplementary material for: A study protocol of a comparative mixed study of the T‐Control catheter
Source: BJUI Compass. 2024 Jan 2;5(3):345–55. doi: 10.1002/bco2.313 (PMC10927921; doi:10.1002/bco2.313)
Supplement: Supplementary file 3 — Data S3. Supporting Information. [file BCO2-5-345-s008.docx]

**START OF THE STUDY**

DATE: ………………………………. PATIENT CODE: ………………………….

1. **PATIENT DATA:**

**AGE:** .................... **SEX:** ........................... **BIRTH DATE:** .................................

**WEIGHT:** ............... **Kg HEIGHT:** ................. **cm**

**Education level:** No studies Primary Secondary University



**Marital status:** Single Married In a relationship Divorced Widow/er



**Coexistence:** I live alone I live as a couple/family



I live with other people who are not from the family nucleus

**User of:** Public healthcare Private healthcare Both

1. **CLINICAL DATA**

**Diagnostic:** ……………………………………………………………………………………………………………

**Urinary catheter need from:** ……………………………………………………………………………………….

**Any clinical symptoms:** ...............................................................................................................................

…………………………………………………………………..............................................................................

**Have you taken any antibiotic treatment in the last two weeks?** YES NO

**Which?** .............................................................................................................................................

**Prior to this study, have you had to use a bladder catheter?** YES NO

**Which?** .............................................................................................................................................

**In case of YES, when did you use the urinary catheter for the last time?**

.......................................................................................................................................................................

**In case of YES, which accessory have you used (urinary bag, plug or valve)?**

.......................................................................................................................................................................

1. **RISK FACTORS AND TOXIC HABITS:**

**SMOKER:** YES NO EX-SMOKER

**In case of YES, number of cigarettes per day:** ..................................................

**In case of EX SMOKER, number of years without smoking:** ...................................................

**ALCOHOL CONSUMPTION:** YES NO OCCASIONALLY

**In case of OCCASIONALLY, specify alcohol consumption:**

1-2 days a week 3-4 days a week 5-6 days a week



On weekends On special occasions (parties, celebrations, etc.)

Others:.............................................................................................................................................................

**SEDENTARY LIFESTYLE:** YES NO

(Sedentary lifestyle is defined as: lack of regular physical activity, defined as: “less than 30 minutes of regular exercise per day and less than 3 days per week).

In case of NO specifying the daily/weekly exercise (check several options if applicable):

1-2 days a week 3-4 days a week 5-6 days a week



On weekends



Low intensity Medium intensity High intensity

Others: ...........................................................................................................................................................

1. **ADVERSE EVENTS DURING THE STUDY RELATED TO THE CATHETER:**

**Has the catheter been accidentally disconnected at some point?** YES NO

If YES, how many times has it been disconnected? ………………………..

What were the reasons for the accidental disconnection?

………………………………………………………………………………………………………………………….………………………………………………………………………………………………………………………….………………

**Has the catheter caused hematuria?** YES NO

If YES, how many times? ………………………..

What were the reasons for the hematuria?

………………………………………………………………………………………………………………………….………………………………………………………………………………………………………………………….………………

**During the catheterization period, has the patient felt pain due to the catheter?** YES NO

If YES, to what degree (on a scale of 1 to 10, with 1 being no pain at all and 10 being a lot of pain)? ……….

How often has he/she felt pain?

………………………………………………………………………………………………………………………….………………………………………………………………………………………………………………………….………………

**Has there been urine loss from the catheter during the study?** YES NO

If YES, how many times? ……………………

What were the reasons for the losses?

………………………………………………………………………………………………………………………….………………………………………………………………………………………………………………………….………………

**Was the catheter clogged during the study?**  YES NO

If YES, how many times? ……………………

What were the reasons for the catheter obstruction?

………………………………………………………………………………………………………………………….………………………………………………………………………………………………………………………….………………

**Has the patient had any other discomfort or limitation due to the catheter?** YES NO

If so, indicate what and what reasons caused them.

………………………………………………………………………………………………………………………….………………………………………………………………………………………………………………………….……………………………………………………………………………………………………………………………………………………………………………………………………………………………………………………………………………………………………………………………………………………………………………………………………………….………………………………………………………………………………………………………………………….………………

1. **DERIVATIVE COSTS DURING THE STUDY:**

**Has the patient attended any hospital emergency visit related to the catheter?** YES NO

In the case of YES, how many visits?………………

What were the reasons?

………………………………………………………………………………………………………….………………………………………………………………………………………………………………………….………………………………

**Has the patient attended any non-hospital emergency visit related to the catheter?** YES NO

In the case of YES, how many visits?………………

What were the reasons?

………………………………………………………………………………………………………….………………………………………………………………………………………………………………………….………………………………

**Has the patient been hospitalised due to the catheter?** YES NO

In the case of YES, how many days? ………………

What were the reasons?

………………………………………………………………………………………………………………………..…………………………………………………………………………………………………………………………….………………**Has the patient had to receive any extra pharmacological treatment related to the catheter?** YES NO

In case of YES, which?……………………………………………………………………

What dose? …………………………………… How long? ………………………..

**Has the patient had to receive any test related to the catheter?** YES NO

In case of YES, which? …………………………………………………………………………………………….………………………….……………………………………………………………………………………………………….………………………….…………**Has the treatment of the patient required any extra material?** YES NO

In case of YES, which and how many? (Ex: diapers, underpads, compresses, etc.)

…………………………………………………………………………………………….………………………….……………………………………………………………………………………………………….………………………….…………**Has the patient required any catheter change?** YES NO

In case of YES, why?

………………………………………………………………………………………………………………………………….

………………………………………………………………………………………………………………………………….

1. **VARIABLES COLLECTED**

| **PATIENT CODE:** |  | | | | | | | | | | **CATHETER LOT:** | | | | | | | | |  | | | | |
| --- | --- | --- | --- | --- | --- | --- | --- | --- | --- | --- | --- | --- | --- | --- | --- | --- | --- | --- | --- | --- | --- | --- | --- | --- |
| **PRESENCE OF INFECTIONS** | | | | | | | | | | | | | | | | | | | | | | | | |
| **Signs/Symptoms related to UTI (urinary tract infection)** | | | | | | Presence  YES / NO / ND  (not determined) | | | | | | | | Observations | | | | | | | | | | |
| Fever? | | | | | |  | | | | | | | |  | | | | | | | | | | |
| Shaking chills? | | | | | |  | | | | | | | |  | | | | | | | | | | |
| Hypotension? | | | | | |  | | | | | | | |  | | | | | | | | | | |
| Presence of any other source of infection? | | | | | |  | | | | | | | |  | | | | | | | | | | |
| Are there symptoms of sepsis? | | | | | |  | | | | | | | |  | | | | | | | | | | |
| **Microorganism detected by URINE CULTURE** | | Presence  YES / NO / ND  (not determined) | | | | | | Quantity  (UFC / ml) | | | | | | Observations | | | | | | | | | | |
| *Escherichia coli*  (specify phylogroup) | |  | | | | | |  | | | | | |  | | | | | | | | | | |
| *Klebsiella* spp.  (specify specie) | |  | | | | | |  | | | | | |  | | | | | | | | | | |
| *Enterobacter* spp. (specify specie) | |  | | | | | |  | | | | | |  | | | | | | | | | | |
| *Serratia* spp.  (specify specie) | |  | | | | | |  | | | | | |  | | | | | | | | | | |
| *Enterococcus* spp. (specify specie) | |  | | | | | |  | | | | | |  | | | | | | | | | | |
| *Proteus* spp.  (specify specie) | |  | | | | | |  | | | | | |  | | | | | | | | | | |
| *Pseudomonas* spp (specify specie) | |  | | | | | |  | | | | | |  | | | | | | | | | | |
| *Acinetobacter* spp. (specify specie) | |  | | | | | |  | | | | | |  | | | | | | | | | | |
| *Candida* spp.  (specify specie) | |  | | | | | |  | | | | | |  | | | | | | | | | | |
| *Staphylococcus* spp. (specify specie) | |  | | | | | |  | | | | | |  | | | | | | | | | | |
| **Other microorganisms identified:** | | | | | | | | | | | | | | | | | | | | | | | | |
| Microorganism  (name, specie) | | | | | Quantity  (UFC / ml) | | | | | | | | Microorganism  (name, specie) | | | | | | | | | | | Quantity  (UFC / ml) |
|  | | | | |  | | | | | | | |  | | | | | | | | | | |  |
|  | | | | |  | | | | | | | |  | | | | | | | | | | |  |
|  | | | | |  | | | | | | | |  | | | | | | | | | | |  |
|  | | | | |  | | | | | | | |  | | | | | | | | | | |  |
|  | | | | |  | | | | | | | |  | | | | | | | | | | |  |
| **Is there a urinary tract infection?** | | | | | | | | YES | | | | | | NO | | | | | | | | Not Determined | | |
| **If there is infection, is it symptomatic or asymptomatic?** | | | | | | | | SYMPTOMATIC | | | | | | | | | | | ASYMPTOMATIC | | | | | |
| **Microorganism detected in CATHETER** | | | Presence  YES / NO / ND  (not determined) | | | | | Quantity  (UFC / ml) | | | | | | | Observations | | | | | | | | | |
| *Escherichia coli* (specify phylogroup) | | |  | | | | |  | | | | | | |  | | | | | | | | | |
| *Klebsiella* spp.  (specify specie) | | |  | | | | |  | | | | | | |  | | | | | | | | | |
| *Enterobacter* spp.  (specify specie) | | |  | | | | |  | | | | | | |  | | | | | | | | | |
| *Serratia* spp.  (specify specie) | | |  | | | | |  | | | | | | |  | | | | | | | | | |
| *Enterococcus* spp. (specify specie) | | |  | | | | |  | | | | | | |  | | | | | | | | | |
| *Proteus* spp.  (specify specie) | | |  | | | | |  | | | | | | |  | | | | | | | | | |
| *Pseudomonas* spp (specify specie) | | |  | | | | |  | | | | | | |  | | | | | | | | | |
| *Acinetobacter* spp. (specify specie) | | |  | | | | |  | | | | | | |  | | | | | | | | | |
| *Candida* spp.  (specify specie) | | |  | | | | |  | | | | | | |  | | | | | | | | | |
| *Staphylococcus* spp. (specify specie) | | |  | | | | |  | | | | | | |  | | | | | | | | | |
| **Other microorganisms identified:** | | | | | | | | | | | | | | | | | | | | | | | | |
| Microorganism  (name, specie) | | | | | Quantity  (UFC / ml) | | | | | Microorganism  (name, specie) | | | | | | | | | | | | | | Quantity  (UFC / ml) |
|  | | | | |  | | | | |  | | | | | | | | | | | | | |  |
|  | | | | |  | | | | |  | | | | | | | | | | | | | |  |
|  | | | | |  | | | | |  | | | | | | | | | | | | | |  |
|  | | | | |  | | | | |  | | | | | | | | | | | | | |  |
|  | | | | |  | | | | |  | | | | | | | | | | | | | |  |
| **TREATMENT WITH ANTIBIOTICS** | | | | | | | | | | | | | | | | | | | | | | | | |
| **Antibiotic** | | Dose | | | | | Duration (start date / end date) | | | | | | | Observations | | | | | | | | | | |
|  | |  | | | | |  | | | | | | |  | | | | | | | | | | |
|  | |  | | | | |  | | | | | | |  | | | | | | | | | | |
|  | |  | | | | |  | | | | | | |  | | | | | | | | | | |
| **ADVERSE EVENTS** | | | | | | | | | | | | | | | | | | | | | | | | |
| **Event** | | | | Presence  YES / NO / ND  (not determined) | | | | | **Event** | | | | | | | | | | | | Presence  YES / NO / ND  (not determined) | | | |
| Accidental spills during insertion | | | |  | | | | | Urine loss per catheter | | | | | | | | | | | |  | | | |
| Accidental disconnection of the catheter | | | |  | | | | | Catheter clogging | | | | | | | | | | | |  | | | |
| Pain associated with catheter | | | |  | | | | | Urethral injury | | | | | | | | | | | |  | | | |
| Hematuria | | | |  | | | | | Hematuria at insertion | | | | | | | | | | | |  | | | |
| Traction hematuria | | | |  | | | | | Hematuria during use | | | | | | | | | | | |  | | | |
| **USE OF SANITARY RESOURCES** | | | | | | | | | | | | | | | | | | | | | | | | |
| **Concept** | | | | | | | | | | | **Catheter used:** | | | | | |  | | | | | | | |
|  |  |  |  |  |  |  |  |  |  |  | Apply (YES / NO) | | | | | | Number (unit) | Cost (€) | | | | | | |
| Catheter | | | | | | | | | | |  | | | | | |  |  | | | | | | |
| Lubricant | | | | | | | | | | |  | | | | | |  |  | | | | | | |
| Gloves | | | | | | | | | | |  | | | | | |  |  | | | | | | |
| Urinary bag | | | | | | | | | | |  | | | | | |  |  | | | | | | |
| Sterile material | | | | | | | | | | |  | | | | | |  |  | | | | | | |
| Additional material (underpads, compresses, etc.) | | | | | | | | | | |  | | | | | |  |  | | | | | | |
| Material to fix the catheter (tape, etc.) | | | | | | | | | | |  | | | | | |  |  | | | | | | |
| Antibiotic treatment | | | | | | | | | | |  | | | | | |  |  | | | | | | |
| Visits to the emergency room or to health centres outside the usual clinical practice related to catheterization | | | | | | | | | | |  | | | | | |  |  | | | | | | |
| Treatments outside routine clinical practice related to catheterization | | | | | | | | | | |  | | | | | |  |  | | | | | | |
| Tests outside routine clinical practice related to catheterization | | | | | | | | | | |  | | | | | |  |  | | | | | | |
| Ambulance transfers | | | | | | | | | | |  | | | | | |  |  | | | | | | |
| Hospitalizations | | | | | | | | | | |  | | | | | |  |  | | | | | | |
| Home visits by health professionals outside normal clinical practice related to catheterization | | | | | | | | | | |  | | | | | |  |  | | | | | | |
| Number of professionals who have participated in the catheterization | | | | | | | | | | |  | | | | | |  |  | | | | | | |
| Healthcare professional time during insertion | | | | | | | | | | |  | | | | | |  |  | | | | | | |
| Others: | | | | | | | | | | |  | | | | | |  |  | | | | | | |

**END OF THE STUDY:**

DATE: ………………………………. PATIENT CODE: ………………………….

**Reason for ending the study:**



The study period has ended.



The patient wants to leave the study.



Complications (specify): ……………………………………………………………………………………...



Others (specify): ………………………………………………………………………………………………….

Follow-up actions after finishing the study, related to catheterization: …………………………………………………………………………………………………………………………………………………………………………………………………………………………………………………………………………………………………………………………………………………………………………………………………………………………………………………………………………………………………………………………………………

Name, signature and data nurse / doctor responsible (with full sheet):
