# Supplementary material for: A study protocol of a comparative mixed study of the T‐Control catheter
Source: BJUI Compass. 2024 Jan 2;5(3):345–55. doi: 10.1002/bco2.313 (PMC10927921; doi:10.1002/bco2.313)
Supplement: Supplementary file 6 — Data S6. Supporting Information. [file BCO2-5-345-s002.docx]

**Patient Experience Questionnaire During the Study (T0)**

**GENERAL DATA**



Date of birth: / /



Sex: Woman Man I prefer not to answer

Why was the bladder catheter prescribed? ……………………………………………

On what date were you catheterised for the first time? ………………………………………………

The first catheter I used was:



Urinary catheter



Bladder catheter with integrated valve



Suprapubic catheter



Suprapubic catheter with integrated valve

I currently use:



Urinary catheter



Bladder catheter with integrated valve



Suprapubic catheter

Suprapubic catheter with integrated valve



I have used:



Urine collection bag



Plugs



Valves



Other:  _____________________

I currently use:



Urine collection bag



Plugs

Valves



Other:  _____________________



The current catheter was inserted at the:



Hospital emergency



Health center emergencies



Outpatient consultations / Urology



Other:  _____________________



Don't know

The catheter insertion procedure was carried out by:



Medical staff



Nursing staff



Other:  _____________________



Don't know

**1. INFORMATION RECEIVED**

**When they catheterized me for the first time they explained it to me clearly and understandably…**

| Totally disagree | |  |  |  |  | Totally agree | |
| --- | --- | --- | --- | --- | --- | --- | --- |
|  |  |  |  |  |  |  |  |
|  | **0** | **1** | **2** | **3** | **4** | **5** |  |
| **…the consequences it would have on my life** |  |  |  |  |  |  |  |
| **…how to handle it** |  |  |  |  |  |  |  |
| **…the different alternatives (plugs, valves or urine collection bag) that could be used, as well as their advantages and disadvantages** |  |  |  |  |  |  |  |
| **…how to use the accessories (plugs, valves or urine collection bag)** |  |  |  |  |  |  |  |

**2. INITIAL SELF-PERCEPTION**



**2.1. When I had this bladder catheter inserted in the hospital, the experience was:**

| **Not painful at all** | 0 | 1 | 2 | 3 | 4 | 5 | 6 | 7 | 8 | 9 | 10 | **Very painful** |
| --- | --- | --- | --- | --- | --- | --- | --- | --- | --- | --- | --- | --- |



**2.2. When I started using this bladder catheter I felt**

**Nothing A lot**

|  | **0** | **1** | **2** | **3** | **4** | **5** |
| --- | --- | --- | --- | --- | --- | --- |
| Sadness |  |  |  |  |  |  |
| Shame |  |  |  |  |  |  |
| Anger |  |  |  |  |  |  |
| Loss of self-esteem |  |  |  |  |  |  |
| Resignation, I have no other choice |  |  |  |  |  |  |
| Relief |  |  |  |  |  |  |
| Security |  |  |  |  |  |  |
| I have gained quality of life |  |  |  |  |  |  |



**3. IMPACT ON LIFESTYLE**

**3.1a. Due to the use of this urinary catheter I have reduced my consumption of water and drinks:**



4 Always



3 Very often



2 Sometimes



1 Occasionally



0 Never

**3.1b. This adaptation of water consumption has negatively affected my daily life**

| **Nothing** | 0 | 1 | 2 | 3 | 4 | 5 | **A lot** |
| --- | --- | --- | --- | --- | --- | --- | --- |



**3.2a. This bladder catheter forces me to adapt my habits (leisure, work, transportation, etc.):**



4 Always



3 Very often



2 Sometimes



1 Occasionally



0 Never

**3.2b. This change in habits has negatively affected my daily life**

| **Nothing** | 0 | 1 | 2 | 3 | 4 | 5 | **A lot** |
| --- | --- | --- | --- | --- | --- | --- | --- |

**3.2c. Please provide some examples if you wish**

**……………………………………………………………………………………………………………….**

**……………………………………………………………………………………………………………….**

| **3.3a Due to this catheter I have been limited in carrying out…** | | | | | | **2.3b. This limitation has negatively affected my daily life** | | | | | | |
| --- | --- | --- | --- | --- | --- | --- | --- | --- | --- | --- | --- | --- |
|  |  |  |  |  |  |  | Nothing A lot | | | | | |
|  | Never (0) | Occasionally(1) | Sometimes (2) | Very Often (3) | Always (4) |  | **0** | **1** | **2** | **3** | **4** | **5** |
| **…daily activities (shopping, housework…)** |  |  |  |  |  |  |  |  |  |  |  |  |
| **…physical activities (running, swimming, walking,…)** |  |  |  |  |  |  |  |  |  |  |  |  |
| **…social activities (family, friends, neighbors,…)** |  |  |  |  |  |  |  |  |  |  |  |  |
| **…my responsibilities (work, child/family care,...)** |  |  |  |  |  |  |  |  |  |  |  |  |



**3.7. I am satisfied with this urinary catheter:**

| **Nothing** | 0 | 1 | 2 | 3 | 4 | 5 | 6 | 7 | 8 | 9 | 10 | **A lot** |
| --- | --- | --- | --- | --- | --- | --- | --- | --- | --- | --- | --- | --- |

**4. USABILITY AND FUNCTIONALITY OF THE CATHETER**



**4.1a. I have stained my clothes because of urine leaking from the catheter.**



4 Always



3 Very often



2 Sometimes

1 Occasionally



0 Never



**4.1b. These leaks have negatively affected my daily life**

| **Nothing** | 0 | 1 | 2 | 3 | 4 | 5 | **A lot** |
| --- | --- | --- | --- | --- | --- | --- | --- |



**4.2a. With the catheter it has been difficult for me to urinate, especially in public bathrooms.**

4 Always



3 Very often



2 Sometimes

1 Occasionally



0 Never



**4.2b. These difficulties have negatively affected my daily life**

| **Nothing** | 0 | 1 | 2 | 3 | 4 | 5 | **A lot** |
| --- | --- | --- | --- | --- | --- | --- | --- |



**4.3a. I feel like people notice that I have the catheter in.**



4 Always



3 Very often



2 Sometimes

1 Occasionally



0 Never



**4.3b. This perception has negatively affected my daily life**

| **Nothing** | 0 | 1 | 2 | 3 | 4 | 5 | **A lot** |
| --- | --- | --- | --- | --- | --- | --- | --- |



**4.4a. I have had to think about buying additional accessories (urine collection bags, plugs,...), whether I have enough or not, if I carry them with me or how to carry them, etc.**

4 Always



3 Very often



2 Sometimes

1 Occasionally



0 Never



**4.4b. This concern has negatively affected my daily life**

| **Nothing** | 0 | 1 | 2 | 3 | 4 | 5 | **A lot** |
| --- | --- | --- | --- | --- | --- | --- | --- |



**4.5a. I have had traction or leakage of urine during physical activity (running, walking, climbing stairs,...)**

4 Always



3 Very often



2 Sometimes

1 Occasionally



0 Never



**4.5b. These tractions or leakages have negatively affected my daily life**

| **Nothing** | 0 | 1 | 2 | 3 | 4 | 5 | **A lot** |
| --- | --- | --- | --- | --- | --- | --- | --- |



**4.6a. I have been worried about water getting into the catheter (when showering, bathing or going to the pool, sea, etc.).**



4 Always



3 Very often



2 Sometimes



1 Occasionally



0 Never

**4.6b. This concern has negatively affected my daily life**

| **Nothing** | 0 | 1 | 2 | 3 | 4 | 5 | **A lot** |
| --- | --- | --- | --- | --- | --- | --- | --- |



**4.7a. I have had problems urinating due to catheter blockages.**



4 Always



3 Very often



2 Sometimes



1 Occasionally



0 Never

**3.7b. These blockages have negatively affected my daily life**

| **Nothing** | 0 | 1 | 2 | 3 | 4 | 5 | **A lot** |
| --- | --- | --- | --- | --- | --- | --- | --- |

**5. DRAINAGE SYSTEM**



**5.1a. The collection bag, cap, valve and/or attachment method has moved or fallen:**

4 Constantly



3 Very often



2 Sometimes



1 Occasionally



0 Never

**5.1b. How much have these movements affected you?**

| **Nothing** | 0 | 1 | 2 | 3 | 4 | 5 | **A lot** |
| --- | --- | --- | --- | --- | --- | --- | --- |



**5.2a. The mechanism to keep the collection bag and/or catheter attached has caused me pain/discomfort:**



4 Constantly



3 Very often

2 Sometimes

1 Occasionally

0 Never

**5.2b. How much has this pain/discomfort affected you?**

| **Nothing** | 0 | 1 | 2 | 3 | 4 | 5 | **A lot** |
| --- | --- | --- | --- | --- | --- | --- | --- |

**5.3a. It has been difficult for me to handle (manipulate, disconnect/connect, open/close) the collection bag, cap or valve of the catheter:**

4 Constantly

3 Very often

2 Sometimes

1 Occasionally

0 Never

**5.3b. How much have these difficulties of use affected you?**

| **Nothing** | 0 | 1 | 2 | 3 | 4 | 5 | **A lot** |
| --- | --- | --- | --- | --- | --- | --- | --- |

**5.4a. The collection bag, cap, valve has given off an unpleasant odor:**

4 Constantly

3 Very often

2 Sometimes

1 Occasionally

0 Never

**5.4b. How much has this smell affected you?**

| **Nothing** | 0 | 1 | 2 | 3 | 4 | 5 | **A lot** |
| --- | --- | --- | --- | --- | --- | --- | --- |

**6. ACTUAL SELF-PERCEPTION**

**6.1. Nowadays the use of the urinary catheter causes me**

**Nothing A lot**

|  | **0** | **1** | **2** | **3** | **4** | **5** |
| --- | --- | --- | --- | --- | --- | --- |
| Sadness |  |  |  |  |  |  |
| Shame |  |  |  |  |  |  |
| Anger |  |  |  |  |  |  |
| Loss of self-esteem |  |  |  |  |  |  |
| Resignation, I have no other choice |  |  |  |  |  |  |
| Relief |  |  |  |  |  |  |
| Security |  |  |  |  |  |  |

**6.2. By wearing this urinary catheter I think I have lost**

**Nothing A lot**

|  | **0** | **1** | **2** | **3** | **4** | **5** |
| --- | --- | --- | --- | --- | --- | --- |
| Freedom |  |  |  |  |  |  |
| Self-esteem |  |  |  |  |  |  |
| Quality of life |  |  |  |  |  |  |
| Perception of my self-image |  |  |  |  |  |  |
| Comfort |  |  |  |  |  |  |
| I haven't lost anything, everything is the same |  |  |  |  |  |  |

**DATA ABOUT YOUR GENERAL HEALTH STATUS**

**Based on the EuroQoL 5D questionnaire (assessment of quality of life in relation to bladder catheterization). Mark with an X in each section the statement that best describes your state of health today:**

**Mobility**

I have no problems walking and/or moving

I have mild problems walking and/or moving

I have moderate problems walking and/or moving

I have severe problems walking and/or moving

I have extreme problems, I can't walk and/or move and I have to stay in bed

**Personal care**

I have no problems with personal care

I have mild problems washing or dressing myself

I have moderate problems washing or dressing myself

I have severe problems washing or dressing myself

I have extreme trouble washing or dressing myself, I'm unable to perform simple self-care tasks

**Everyday activities** (such as work, study, housework, family activities, or leisure time)

I have no problems doing my daily activities

I have mild problems doing my daily activities

I have moderate problems doing my daily activities

I have severe problems performing my daily activities

I have extreme problems, I am unable to carry out my daily activities

**Pain / Discomfort**

I have no pain or discomfort

I have minor pain or discomfort

I have moderate pain or discomfort

I have severe pain or discomfort

I have extreme pain or discomfort

**Anxiety / Depression**

I'm not anxious or depressed

I am mildly anxious or depressed

I am moderately anxious or depressed

I am severely anxious or depressed

I am extremely anxious or depressed

**To help describe how good or bad your health is, we have drawn a scale similar to a thermometer in which 100 is the best health you can imagine, and 0 is the worst health you can imagine.**

**Please draw a line from the box that says “your health status today,” to the point on the scale that you think indicates how good or bad your health status is today.**
