# Supplementary material for: A study protocol of a comparative mixed study of the T‐Control catheter
Source: BJUI Compass. 2024 Jan 2;5(3):345–55. doi: 10.1002/bco2.313 (PMC10927921; doi:10.1002/bco2.313)
Supplement: Supplementary file 7 — Data S7. Supporting Information. [file BCO2-5-345-s001.docx]

**CATHETER INSERTION**

Please rate the following statements by writing down a value between 1 and 5 in the corresponding box based on the following assessments:

1 Strongly disagree

2 Disagree

3 Neutral, neither agree nor disagree

4 Agree

5 Totally agree

If you have performed the catheterisation with the Foley catheter please rate the affirmations in the first column, if you have performed the catheterisation with the T-Control catheter, please rate the affirmations in the second column. If you have performed the catheterisation with both devices please rate in both columns.

| **Affirmations** | **Foley catheter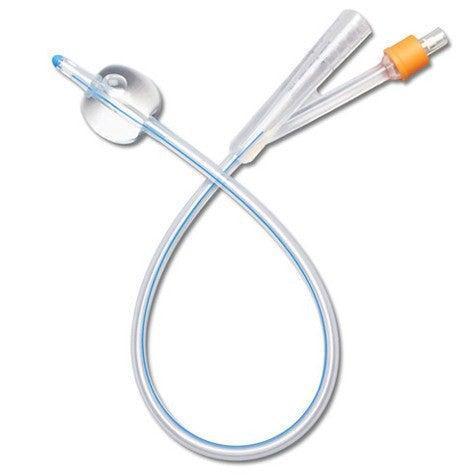** | **T-Control 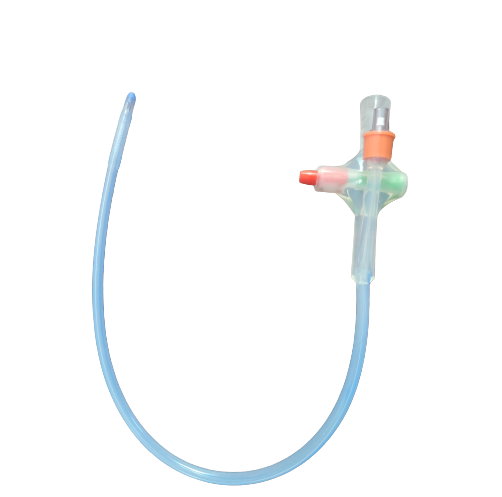** |
| --- | --- | --- |
| I found the device comfortable during insertion. |  |  |
| I found the device easy to use. |  |  |
| I believe the device prevents urine leakage during insertion. |  |  |
| I have had no difficulties in maintaining the sterility of the process. |  |  |
| The collection of urine sample for culture has been easy for me. |  |  |
| There is an increased risk of accidental urine leakage after insertion (unintentional opening, accidental disconnection...). |  |  |
| During the insertions, I have had some leakage. |  |  |
| The insertion of the catheter has been stressful for me. |  |  |
| It would be much better off inserting the catheter with the help of another person. |  |  |
| In general, I have felt comfortable using the device. |  |  |
| If given the choice in the future, I would choose this catheter for my patients. |  |  |
| The procedure was easy for me. |  |  |

**NASA – TLX QUESTIONNAIRE**

Please note in the appropriate box how you would rate the following questions about workload when a catheter is inserted. Enter a value from 1 to 10, with 1 being the lowest score and 10 being the highest score.

If you have performed the catheterisation with the Foley catheter please rate the affirmations in the first column, if you have performed the catheterisation with the T-Control catheter, please rate the affirmations in the second column. If you have performed the catheterisation with both devices please rate in both columns.

| **Affirmations** | **Foley catheter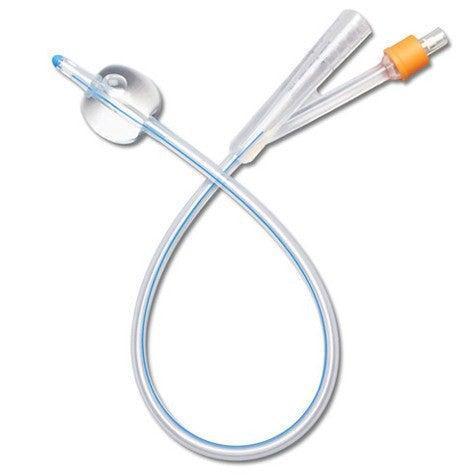** | **T-Control 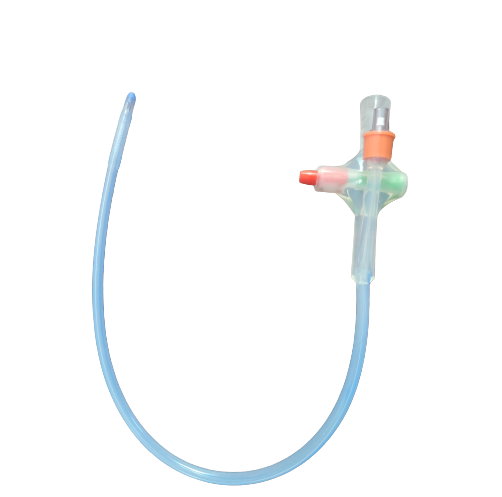** |
| --- | --- | --- |
| 1. **Mental demand**. How much mental activity is required to perform a bladder catheterisation (thinking, deciding, remembering or searching)? |  |  |
| 1. **Physical demand**. How much physical activity is required to perform the bladder catheterisation (push, pull, turn, control, activate)? |  |  |
| 1. **Temporary demand**. How much pressure do you feel due to time, speed, or the rate at which tasks are performed? |  |  |
| 1. **Effort**. How much work effort (mental and physical) do you have to do to achieve your level of performance during the bladder catheterisation? |  |  |
| 1. **Performance.** What level of satisfaction do you have with your performance after bladder catheterisation? |  |  |
| 1. **Level of frustration.** How insecure, discouraged, irritated, stressed, and upset versus confident, satisfied, happy, relaxed, and complacent do you feel about having a bladder catheter**?** |  |  |

**OTHER COMMENTS**

Please write in the following any comments or suggestions that you would like to add.

……………………………………………………………………………………………………………………………………………………………………………………………………………………………………………………………………………………………………………………………………………………………………………………………………………………………………………………………………………………………………………………………………………………………………………………………

**COMPARATIVE STUDY**

In the event that you have been able to perform catheterisations with both bladder catheters, a conventional **Foley catheter** and a **T-Control® catheter**, mark with an X the device that seems most compatible with the statement.

| **Affirmations** | **Foley catheter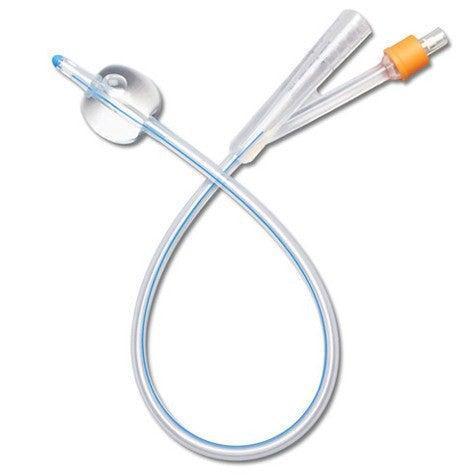** | **T-Control 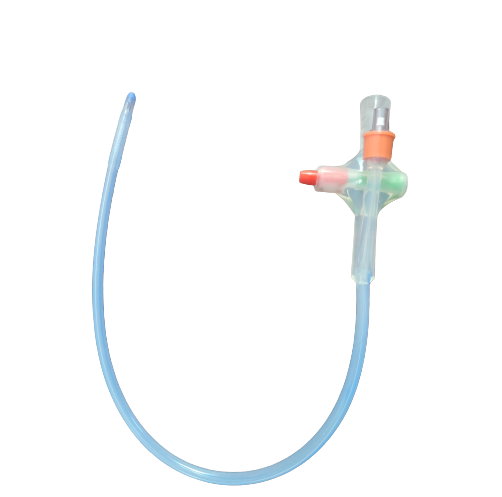** |
| --- | --- | --- |
| It is the most comfortable for insertion. |  |  |
| Urine sample collection is easier. |  |  |
| Prevents urine leakage during insertion. |  |  |
| Prevents urine leakage after insertion. |  |  |
| I have had no difficulties in maintaining the sterility of the process. |  |  |
| It allows me more time to think about the steps to follow during the procedure. |  |  |
| There is an increased risk of accidental urine leakage after insertion (unintentional opening, accidental disconnection...). |  |  |
| The insertion of the catheter has been more stressful for me. |  |  |
| I would be more likely to do well without help. |  |  |
| In general, I have felt comfortable using the device. |  |  |
| If given the choice in the future, I would choose this catheter for my patients. |  |  |

**FINAL ASSESSMENT**

Please write in the following any comments or suggestions that you would like to add.

………………………………………………………………………………………………………………………………………………………………………………………………………………………………………………………………………………………………………………………………………………………………………………………………………………………………………………………………………………………………………………………………………………………………………………………………………………………………………………………………………………………………………………

………………………………………………………………………………………………………………………………………………………………………………………………………………………………………………………………………………………………………………………………………………………………………………………………………………………………………………………………………………………………………………………………………………………………………………………………………………………………………………………………………………………………………………
