# Supplementary material for: A study protocol of a comparative mixed study of the T‐Control catheter
Source: BJUI Compass. 2024 Jan 2;5(3):345–55. doi: 10.1002/bco2.313 (PMC10927921; doi:10.1002/bco2.313)
Supplement: Supplementary file 8 — Data S8. Supporting Information. [file BCO2-5-345-s006.docx]

**PATIENT INFORMATION SHEET**

**STUDY TITLE:** Comparative mixed study of the Foley-type catheter with the T-Control® catheter in patients with long-term catheterization

**STUDY CODE:** RM-TCONTROL-2022-02

**DATE:** 14th of February of 2023.

**VERSION NUMBER:** v1.0

**PRINCIPAL INVESTIGATOR:** Santiago Pérez García, nursing research and teaching unit.

**CENTRE:** 12 de Octubre University Hospital

**INTRODUCTION**

We are writing to inform you about a research study, in which you are invited to participate. The study has been approved by the Research Ethics Committee of Hospital Doce de Octubre in accordance with current legislation, the Biomedical Research Law of July 3, 2007.

Our intention is only that you receive the correct and sufficient information so that you can evaluate and judge if you want to participate in this study. To do this, read this information sheet carefully and we will clarify any doubts that may arise after the explanation. In addition, you can consult with the people you consider appropriate.

**VOLUNTARY PARTICIPATION**

You should know that your participation in this study is voluntary and that you can decide not to participate or change your decision and withdraw your consent at any time, without altering your relationship with your doctor or causing any harm to your treatment.

**DESCRIPTION OF THE STUDY**

The study lasts four weeks and seeks to determine the efficacy of the T-Control® bladder catheter, a catheter under study that has an integrated valve at its distal end, compared to a conventional Foley-type catheter, commonly used in clinical practice. To do this, the rate of urinary infection, and adverse effects such as catheter disconnection, obstruction and pain will be evaluated. Likewise, we intend to know your experience with the urinary catheter in terms of comfort, satisfaction and quality of life.

To do this, at the time of changing the usual catheter, a urine sample will be collected to perform a urine culture and thus determine the presence or absence of a urine infection, recording the results anonymously. Likewise, at this time you will receive information about the device with which you have been randomly catheterized and you will be given a diary for the collection of relevant information for the study.

Four weeks after the catheter insertion, you will be called for a follow-up visit, in which the information collection diary will be collected, a urine sample will be taken to evaluate the presence of urinary tract infections. During this follow-up visit, you will be asked to complete a questionnaire related to the study, as well as background information that may be related to it, and you may be invited to participate in a discussion group with other patients to evaluate the experience with the catheter. management difficulties and degree of satisfaction.

It is guaranteed that the samples and data associated with the study will be kept under security conditions and that the subjects cannot be identified by persons other than those authorized. Likewise, it is guaranteed that the urine samples will be eliminated by the usual procedure of the microbiology service, without any personal data appearing in it.

**BENEFITS AND RISKS ARISING FROM YOUR PARTICIPATION IN THE STUDY**

It is possible that you will not obtain any benefit for your health from participating in this study, however, it is expected that the study will contribute to improving health, with the prevention of infections and personal well-being, providing greater quality of life, autonomy and comfort among the people who use indwelling urinary catheters.

By agreeing to participate in this study you will be responsible for complying with the study activities. No risks other than those inherent to any bladder catheterization, obtaining urine samples, or derived from your participation in the study are foreseen; but if you detect any adverse situation, you agree to notify the main researcher.

The follow-up visit may take longer than usual in normal clinical practice due to the completion of the corresponding questionnaire.

**ETHICAL CONSIDERATIONS**

This study will be carried out in accordance with the recommendations established in the Declaration of Helsinki and the Standards of Good Clinical Practice and its exclusive purpose is to evaluate the safety and effectiveness of the new T-Control® catheter. This means that by participating in this study, you will not undergo any unnecessary procedures, nor will you stop receiving the care necessary to treat your illness if you decide not to participate.

**CONFIDENTIALITY**

The promoter and researchers undertake that their personal data will be treated confidentially and will be processed in accordance with current regulations contained in Organic Law 3/2018, on the Protection of Personal Data and guarantee of digital rights and the Regulation (EU) 2016/679 of the European Parliament and of the Council of April 27, 2016 on Data Protection (GDPR).

The data collected for the study will be identified by a code so that it does not include information that could identify you, and only the principal investigator and collaborating researchers will be able to relate said data to you and your medical history. Therefore, your identity will not be revealed to anyone except in cases of medical emergency or legal requirement. The treatment, communication and transfer of personal data of all participants will comply with the provisions of this law.

Access to your identified personal information will be restricted to the main researcher, collaborating researchers, health authorities, the Research Ethics Committee and authorized personnel; when necessary to verify the data and procedures of the study, but always maintaining their confidentiality in accordance with current legislation.

The data will be collected in a research file under the responsibility of the center and will be processed solely and exclusively within the framework of your participation in this study.

Under no circumstances will you be personally identified in written publications or scientific presentations where the results of the study are presented.

If you request it, you may be provided with information about the analyzes to which your samples have been subjected during the clinical trial, as well as its results.

In accordance with what is established by data protection legislation, you can exercise the rights of access, modification, opposition and cancellation of data, for which you must contact the principal investigator of the study.

**LIABILITY INSURANCE**

The promoter has contracted an insurance that covers the Civil Liability that is required as a result of the damages and losses caused involuntarily to the participants, up to a maximum of €350,000.00 per patient.

**OTHER RELEVANT INFORMATION**

If you decide to withdraw consent to participate in this study, no new data will be added to the database, but data that has already been collected will be used. To do this, you should contact Santiago Pérez García, principal investigator of the Research and Teaching Unit, or Jose Medina Polo, doctor of the urology service, collaborating researcher, both from the 12 de Octubre University Hospital.

By signing the attached consent form, you agree to comply with the study procedures outlined to you.

**INFORMED CONSENT**

**STUDY TITLE:** Comparative mixed study of the Foley-type catheter with the T-Control® catheter in patients with long-term catheterization

**STUDY CODE:** RM-TCONTROL-2022-02

**DATE:** 14th of February of 2023.

**VERSION NUMBER:** v1.0

I, Mr./Ms. ,………………………………………………………… (name and surname of the father/mother or guardian), residing at ...................................................................................and ID number.................... ...................... I declare that:

I have read the information sheet that was given to me.

I have been able to ask questions about the study.

I have received enough information about the study.

I have spoken with:

..............................................................................................

(name of researcher)

I hereby freely agree to participate in the study and give consent for the use of my data.

I understand that my participation is voluntary.

I understand that I can withdraw from the study voluntarily, without having to give reasons and without this affecting my healthcare.

**Patient’s signature: Researcher’s signature:**

**Name: Name:**

**Date: Date:**
